# A high fiber diet or supplementation with *Lactococcus lactis* subspecies cremoris to pregnant mice confers protection against intestinal injury in adult offspring

**DOI:** 10.1080/19490976.2024.2337317

**Published:** 2024-04-15

**Authors:** Maria E. Barbian, Joshua A. Owens, Crystal R. Naudin, Patricia Denning, Ravi M. Patel, Rheinallt M. Jones

**Affiliations:** a Department of Pediatrics, Emory University School of Medicine and Children’s Healthcare of Atlanta, Atlanta, USA; b Department of Biology, Lipscomb University, Nashville, USA; c Department of Medicine, Emory University School of Medicine, Atlanta, USA; d Department of Pediatrics, Emory University School of Medicine, Atlanta, USA

**Keywords:** Butyrate, probiotics, antenatal diet, pregnancy, diet, colitis, DSS

## Abstract

The diet during pregnancy, or antenatal diet, influences the offspring’s intestinal health. We previously showed that antenatal butyrate supplementation reduces injury in adult murine offspring with dextran sulfate sodium (DSS)-induced colitis. Potential modulators of butyrate levels in the intestine include a high fiber diet or dietary supplementation with probiotics. To test this, we supplemented the diet of pregnant mice with high fiber, or with the probiotic bacteria *Lactococcus lactis* subspecies cremoris or *Lactobacillus rhamnosus* GG. We then induced chronic colitis with DSS in their adult offspring. We demonstrate that a high fiber antenatal diet, or supplementation with *Lactococcus lactis* subspecies cremoris during pregnancy diminished the injury from DSS-induced colitis in offspring. These data are evidence that antenatal dietary interventions impact offspring gut health and define the antenatal diet as a therapeutic modality to enhance offspring intestinal health.

## Introduction

The fetal environment plays a dynamic role in fetal growth and development and influences the offspring’s long-term health through developmental programming.^1^ Through pre-clinical and clinical studies, our understanding of the influence of the diet during pregnancy (antenatal diet) on the offspring’s health has expanded.^2^ For example, it was reported in mice that a high fat diet during pregnancy causes an expansion of Firmicutes in the offspring’s gut microbiome and increases the offspring’s susceptibility to neonatal intestinal inflammation.^3^ Another study in mice reported that a diet rich in the aryl hydrocarbon receptor ligand indole-3-carbinol, prevented the development of necrotizing enterocolitis in neonatal mice.^4^ Attesting to clinical relevance, several studies in humans have corroborated the importance of the antenatal diet on the offspring’s health.^5–9^

Recently, our research group evaluated the effect of antenatal butyrate supplementation to pregnant mice on the intestinal health of their offspring.^10^ Butyrate is a short-chain fatty acid (SCFA) produced by the gut microbiome through the fermentation of undigested dietary fiber.^11^ Butyrate influences intestinal health through enhancement of the intestinal barrier,^12^ down regulation of intestinal inflammation^13^ and regulation of intestinal immune response.^12^ Supplementation of the antenatal diet with butyrate reduced injury in adult offspring in a model of dextran sodium sulfate (DSS) induced colitis, with associated reduction in pro-inflammatory gene expression.^10^ Supplementing the antenatal diet with butyrate also resulted in shifts in the adult offspring’s gut microbiome composition with increased prevalence of *Bifidobacterium*, and increased alpha diversity.^10^

Based on these findings, we sought to evaluate whether supplementing the antenatal diet to increase endogenous butyrate levels would also reduce injury from colitis in adult offspring. Butyrate production by the microbiome may be increased through a high fiber diet (HFD),^14,15^ or by dietary supplementation with lactic acid bacteria, where bacteria produce lactate that is subsequently converted to butyrate by gut microbes that harbor a gene coding for *butyryl-CoA:acetate CoA-transferase*.^16,17^ Our research group identified the novel probiotic *Lactococcus lactis* subsp. cremoris (LLC) as being highly efficacious in suppressing colitis-induced gut injury.^18,19^ Herein, we report that a high fiber antenatal diet, or supplementation of the antenatal diet with LLC lowered colitis disease activity in offspring, and propose that supplementation of the antenatal diet with interventions that enhance butyrate generation may be an effective therapeutic strategy to enhance gut health in offspring.

## Methods

### Animal studies

Experiments were performed using wild-type C57BL/6 mice (Jackson Laboratories, Bar Harbor, ME). The mice were bred at the Department of Animal Resources facility at Emory University. Animal procedures were approved by the Institutional Animal Care and Use Committee at Emory University.

### Bacterial strains and culture preparation

Probiotic bacteria purchased from the American Type Culture Collection (ATCC) (Manasas, VA) included *Lactococcus lactis* subspecies cremoris ATCC 19,257 (LLC) and *Lactobacillus rhamnosus* GG ATCC 53,103 (LGG). All media were propagated according to ATCC instructions. Probiotics were suspended in hanks balanced salt solution (HBSS) to a dose of 1 × 10^9^ colony forming units, which has been reported to be beneficial to murine intestinal health.^19,20^

### Murine diets

The mice received chow *ad libitum*. Breeding pairs received either a regular diet, a high fiber diet (HFD), LLC or LGG during pregnancy. The regular diet used was 5053-PicoLab® Rodent Diet 20, 5053 6% Fiber (LabDiet Inc, MO). The high fiber diet (HFD) used was isocaloric Modified AIN-93 G Purified Rodent Diet with 30% Fiber (15% wt:wt) with a 30% soluble fiber:70% cellulose ratio (Dyets Inc., PA, Lot #7284–5).

### Mating pairs and experimental groups

Breeding pairs of mice (one male and one female) were randomly assigned to a diet which they received during pregnancy. In each experiment, mice originated from the same cage, ensuring that the microbiome composition was homogenous between all groups at the beginning of the experiment. The experimental groups included dietary variables fed to female mice before conception and during pregnancy, including: HFD, daily LLC supplementation via oral gavage, daily LGG supplementation via oral gavage, LLC with HFD, or regular diet (control). The mating pairs which did not receive probiotic supplementation received daily oral gavage of HBSS. Within 24 h of birth, all mating pairs were placed on a regular diet and did not receive any further dietary supplementation. The offspring of all mating pairs were weaned at 3 weeks of age based on their sex and experimental group and received a regular diet. For each experimental variable, two to three breeding pairs of mice generated all the offspring for each specific group.

### DSS-induced colitis

Chronic colitis was induced in offspring at 6–8 weeks of age through administration of DSS (Thermo Scientific, Fair Lawn, NJ, Lot # 187742 and Lot # 207466), established previously as a murine model of colitis.^20,21^ To induce colitis, DSS was dissolved in the drinking water of the mice. The mice received DSS for 2 cycles. First, they received 2% DSS for 7 days, followed by 7 days of recovery with no DSS. Last, they received another 7 days of 2% DSS. The degree of colitis was evaluated by using a disease activity index (DAI) scoring tool, colon length measurement at the time of sacrifice and histomorphological injury scoring. DAI scoring is an established method to measure the degree of colitis in models of DSS colitis; it accounts for stool consistency, presence of blood in the stool and percent weight loss of each mouse.^22^ DAI scores range from 0 to 12 and the score is proportional to the degree of intestinal injury with a score of ‘0’ being no injury, and a score of ‘12’ indicating severe colitis. DAI scoring began at day 3 of DSS colitis model. Colon length is another measure of colonic injury, as colitis causes contraction and shortening of the colon.^23^ Thus, colon length is inversely related to the severity of colitis and was measured at sacrifice.

### Hematoxylin-eosin staining and scoring of tissue for intestinal injury

Following completion of the chronic colitis model, the murine offspring were sacrificed, and their colon was harvested. Each colon was fixed in 4% formalin, dehydrated, paraffin embedded and then sectioned. The sections underwent deparaffinization, hydration in alcohol gradient, and staining with Hematoxylin and Eosin (H&E) by Emory Winship Pathology Core. Images were scored for the degree of colitis using a 2-tiered scoring system, adapted from an established scoring guideline.^24^ The histologic activity index (HAI) score evaluates the degree of inflammatory cell infiltration and the integrity of the mucosal architecture. HAI score ranges from 0 to 6 and is proportional to the degree of injury. Inflammatory cell infiltration is considered mild, moderate, or severe based on the degree of inflammatory cells in the colonic tissue. Inflammatory cell infiltration to the level of the mucosa is considered mild (score of 1), to the mucosa and submucosa is considered moderate (score of 2) and transmural inflammatory cell infiltration is considered severe (score of 3). Next, the extent of epithelial injury is determined based on architectural epithelial changes such as focal erosions (score of 1), erosions with focal ulcerations (score of 2) or extended ulcerations with or without granulation tissue or pseudo polyps (score of 3). The HAI score is calculated as a sum of the inflammatory cell infiltrate score (0–3) and epithelial architecture score (0–3). Sections were scored in blinded fashion.

### Statistics

Statistical significance was determined by Student’s *T* test for paired samples and one-way ANOVA for data with more than 2 groups using Prism 8 (GraphPad, San Diego, CA). A p-value of <0.05 was defined as statistically significant. All data are presented as mean ± standard error of the mean (SEM).

## Results

### A high fiber antenatal diet in mice reduces DSS-induced chronic colitis injury in adult offspring

**Figure 1.**
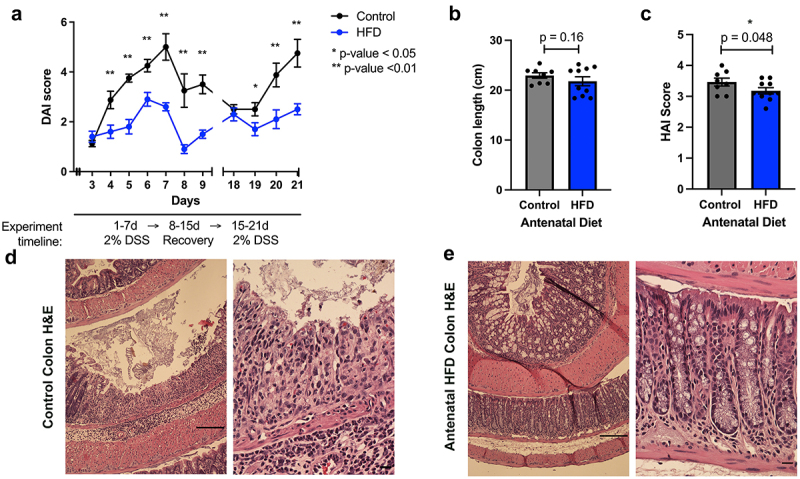
Antenatal high fiber diet reduces injury from DSS colitis in adult murine offspring.

At 6 weeks of age, offspring mice from dams that received an HFD or control regular diet were subjected to a model of DSS-induced chronic colitis. Health monitoring during the chronic colitis model revealed that offspring from HFD supplemented mothers had significantly lower DAI compared to offspring from control diet fed mothers (Figure 1(a)). For example, at day 7, the last day of the first cycle of DSS, the mean DAI score for the control group was 5 ± 1.5, whereas the mean DAI for the HFD group was 2.6 ± 0.5 (*p* < 0.01). At day 21 of the experiment, the final day of the second cycle of DSS, the mean DAI score for the control group was 4.8 ± 1.6, whereas the mean DAI for the HFD group was 2.5 ± 0.7 (*p* < 0.01). No significant differences in colon lengths were detected between the two groups (*p* = 0.16) (Figure 1(b)), but histopathologic analysis revealed antenatal HFD offspring had lower injury scores than control group offspring with HAI scores of 3.1 ± 0.3 for the antenatal HFD offspring and HAI scores of 3.5 ± 0.4 for the control group offspring (*p* = 0.048) (Figure 1(c–e)). In each of our experimental group, we did not detect a sex-specific effect. Together, these data show that an antenatal HFD can elicit enduring cytoprotective effects in the intestine of murine offspring up to 9 weeks of age.

(a) Quantification of intestinal injury through DAI score in 6-week-old C57BL/6 mice subjected to chronic 2% DSS-induced colitis model. Experimental groups include offspring from mothers on a HFD antenatal diet (*n* = 10), and offspring from mothers on a control antenatal diet (*n* = 8). DAI score ranges from 0 to 6 and is proportional to the degree of intestinal injury. Student’s t-test. *p < 0.05, **p < 0.01. (b) Colon length of mice described in (a) measured at the time of sacrifice (9 weeks-old). (c) HAI score quantifies colonic injury by a validated scoring tool, the score ranges from 0 to 6 and is proportional to the degree of colonic injury. Student’s t-test. *p < 0.05). (d) Representative H&E images of proximal colonic tissue in mice in antenatal control group. (e) Representative H&E images of proximal colonic tissue in mice in antenatal HFD group. Scale bar is 50μm.

### *Antenatal supplementation of* L. lactis cremoris *(LLC) reduces DSS-induced chronic colitis injury in adult offspring*

**Figure 2.**
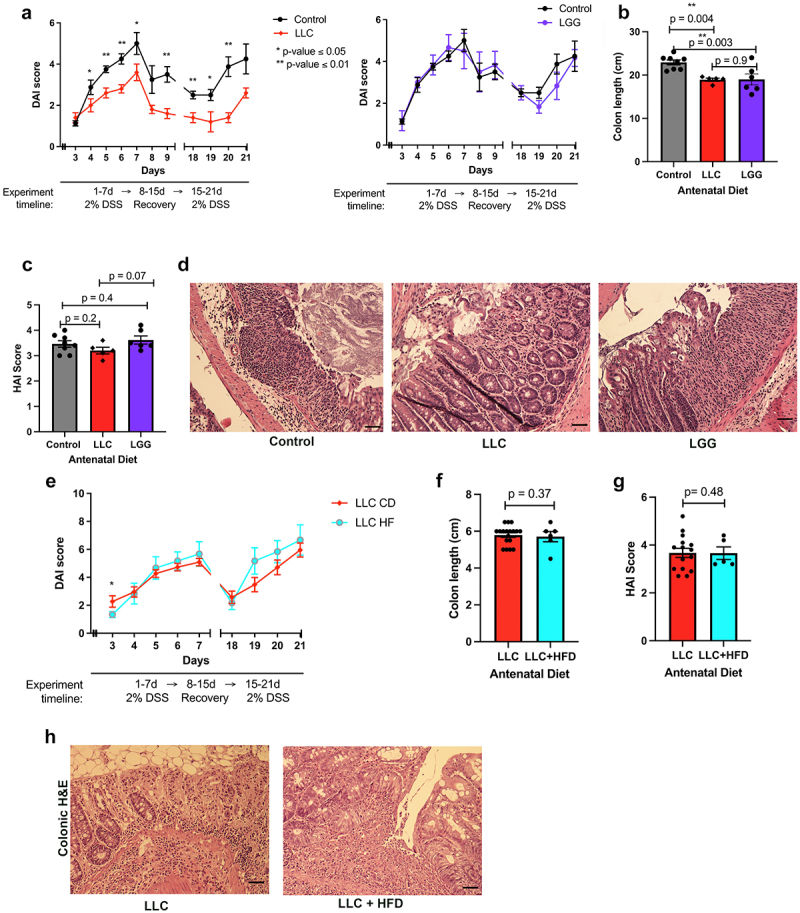
Supplementation of the antenatal diet with probiotics impacts the extent of DSS-induced colitis injury in adult offspring.

At 6 weeks of age, offspring mice from dams that were supplemented LLC, LGG or control group were subjected to a model of DSS-induced chronic colitis. The offspring from the antenatal LLC supplemented group exhibited significantly lower DAI scores compared to the offspring from the control groups (Figure 2(a)). At day 7, the mean DAI score for the control group was 5 ± 1.5, whereas mean DAI for the LLC group was 3.6 ± 0.9 (*p* = 0.04). On day 21 of DSS, the mean DAI score for the control group was 4.8 ± 1.6, versus the mean DAI for the LLC group was 2.6 ± 0.5 (*p* < 0.01). By contrast, the DAI scores of the offspring in the antenatal LGG group were not significantly different from the DAI scores of offspring in the control group (Figure 2(a)). Specifically, at day 7, the mean DAI score for the control group was 5 ± 1.5, and the mean DAI for the LGG group was 4.5 ± 2 (*p* = 0.3). On day 21 of DSS, the mean DAI score for the control group was 4.8 ± 1.6, versus the mean DAI for the LGG group was 4.2 ± 1 (*p* = 0.2). Control group offspring had significantly longer colons (22.9 ± 1.6 cm) than the offspring from the antenatal LLC (18.9 ± 0.8 cm) and LGG (19 ± 3.1 cm) supplemented groups (*p* < 0.004 and *p* < 0.003, respectively) (Figure 2(b)). Despite lower DAI scores, histopathologic grading (HAI) to assess the intestinal injury did not show a significant difference in the level of injury between the control group and the antenatal LLC group (*p* = 0.21), or the control group and the antenatal LGG group (*p* = 0.43) (Figure 2(c, d)). However, we did detect lower HAI score in the antenatal LLC supplemented group compared to the LGG group, with a mean HAI score of 3.2 ± 0.3 in the antenatal LLC group and a mean HAI score of 3.6 ± 0.4 in the antenatal LGG group (*p* = 0.07) (Figure 2(c, d)). These data show that supplementation of the antenatal diet with LLC, but not LGG, conferred cytoprotection in the intestine of 9-week-old murine offspring.

(a) Quantification of intestinal injury through DAI score on 6-week-old C57BL/6 mice subjected to chronic 2% DSS-induced colitis model. Experimental groups include offspring from mothers whose antenatal diet was supplemented daily with 1 × 10^9^ CFU of *Lactococcus lactis* subsp. Cremoris (LLC) (*n* = 5), *Lactobacillus rhamnosus* GG (LGG) (*n* = 6), or with equal volume of HBSS (control) (*n* = 8). DAI score ranges from 0 to 6 and is proportional to the degree of intestinal injury. Student’s t-test. * *p* < 0.05, ** *p* < 0.01. (b) Colon length of mice described in (a) measured at the time of sacrifice (9 weeks-old). One-way ANOVA. ** *p* < 0.01. (c) HAI score quantifies colonic injury by a validated scoring tool, the score ranges from 0 to 6 and is proportional to the degree of colonic injury. One-way ANOVA. (d) Representative H&E images of proximal colonic tissue in mice described in (a). Scale bar is 50μm. (e) Quantification of intestinal injury through DAI score on 6-week-old C57BL/6 mice subjected to chronic 2% DSS-induced colitis model. Experimental groups include offspring from mothers on a HFD antenatal and supplemented daily with LLC (LLC HF) (*n* = 6), or on a control diet supplemented daily with LLC (*n* = 18) (LLC). Student’s t-test. (f) Colon length of mice described in (e) measured at the time of sacrifice (9 weeks-old). Student’s t-test. (g) HAI score quantifies colonic injury by a validated scoring tool, the score ranges from 0 to 6 and is proportional to the degree of colonic injury. Student’s t-test. (h) Representative H&E images of proximal colonic tissue in mice described in (f). Scale bar is 50μm.

Given the reduction in intestinal injury among adult offspring born to dams with antenatal HFD or LLC supplementation during pregnancy, we evaluated if a combination of LLC supplementation and a HFD during pregnancy would work in synergy to improve gut cytoprotection in offspring. Adult offspring from antenatal HFD with LLC or antenatal LLC supplementation were exposed to DSS-induced chronic colitis. Analysis revealed that there was no further reduction in intestinal injury in offspring whose mothers were fed antenatal HFD and supplemented with LLC, compared to offspring from mothers with antenatal LLC supplementation (Figure 2(e–h)). DAI scores were not significantly different between the two groups. On day 7 of DSS, the mean DAI score for the LLC group was 5 ± 1.2, and the mean DAI for the LLC with HFD group was 5.6 ± 2.2 (*p* = 0.2). On day 21 of DSS, the mean DAI score for the LLC group was 5.9 ± 2.3, versus the mean DAI for the LLC with HFD group was 6.7 ± 2.6 (*p* = 0.3). There was also no significant difference in colon length or HAI scores between the two groups, with mean HAI scores of 3.7 ± 0.7 in the LLC group and 3.6 ± 0.6 in the HFD with LLC group (*p* = 0.48).

### Discussion

In this report, we investigated the effects of antenatal dietary supplementation with a HFD, or LLC and LGG probiotics on the adult offspring’s response to experimentally induce chronic colitis. This study corroborates previous findings that the antenatal diet influences the intestinal health of the offspring.^3,4,10^ Given our previous findings that antenatal butyrate supplementation in mice leads to a reduction in gut injury in offspring exposed to models of DSS colitis, we anticipated that antenatal supplementation with HFD would also reduce injury in DSS-colitis among adult offspring, as supplementation with HFD has been reported to increase butyrate production by the microbiome.^11^ Indeed, our results highlight that a HFD during pregnancy led to a significant reduction in gut injury in adult offspring exposed to DSS colitis, with a significant reduction in colitis by DAI and HAI score. Interestingly, the lowering of DAI and HAI by HFD during pregnancy did not impact colon length, which is often used as a faithful parameter to quantitate the extent of gut DSS-induced injury.

In addition, we found that supplementation of the antenatal diet with LLC was associated with a reduction in intestinal injury in the adult offspring compared to supplementation of the antenatal diet with LGG, evinced by lower DAI scores. We also evaluated whether supplementing the antenatal diet with a HFD and LLC would be more protective to the offspring than LLC alone. However, HFD and LLC did not engender a cumulative cytoprotective effect. We note that the HFD combined with LLC experiment outlined in Figure 2(e–h) was done with a different batch of DSS reagent than the experiments outlined in Figure 2(a–d), and therefore DAI scores could not be directly compared. Nevertheless, the data intriguingly demonstrate that the LLC probiotic, but not LGG was effective in conferring protection in offspring.

A description of the mechanism whereby a HFD or dietary supplementation with LLC to pregnant dams transmits beneficial influences to offspring is currently elusive. We speculate that one possible mechanism is via the generation of elevated levels of SCFAs within the pregnant dams. Indeed, a HFD^14,15^ or dietary supplementation with lactic acid bacteria^16,17^ has been reported to increase levels of SCFAs in gut tissue and serum. However, measurement of butyrate concentrations in gestating pups within the uterus at day E19, or in the gut tissue of 2-week-old offspring did not detect any significant differences in SCFAs levels, even in E19 pups or 2-week-old offspring from dams directly supplemented with butyrate during gestation. These observations support the notion that the E19 pups are not directly exposed to elevated butyrate and that the 2-week-old offspring do not themselves generate elevated butyrate levels in their microbiome. These observations may suggest that the elevated cytoprotective states in offspring may be due to microbiome transfer from dams to offspring and that the inherited microbiome contains a selection of beneficial microbes. It is also possible that gestating pups may be subject to epigenetic changes in utero since fetal development constitutes the most active period for epigenetic DNA imprinting. Given butyrate’s activity as an HDAC inhibitor, it is plausible that part of antenatal butyrate’s protection against intestinal injury in offspring is due to inherited epigenetic effects on genes within the offspring’s intestinal epithelium. As of the publication of this manuscript, the mechanism whereby an HFD or dietary supplementation with LLC to pregnant dams transmits beneficial influence to offspring remains elusive, although it is the subject of intense scrutiny within our research group.

Together, these data suggest that antenatal HFD provides the most significant and consistent protection against intestinal injury in adult offspring exposed to DSS-induced chronic colitis. Antenatal LLC also reduced injury in adult offspring; however, this reduction in injury was not as consistent with HFD, with reduction in DAI but not HAI. Interestingly, antenatal LGG did not induce any reduction in intestinal injury in adult offspring. We postulate that these findings are related to the amount of butyrate produced from each of these diets, with enhanced butyrate production in the HFD and LLC diets. Additionally, since the combination of HFD and LLC supplementation did not provide additional cytoprotection, this suggests that there may be a limit to the amount of butyrate that induces protection or the amount of butyrate produced by our gut microbiome. Thus, a high fiber diet or LLC probiotic when consumed during pregnancy may provide therapeutic options to enhance offspring intestinal health.

## Conclusion

These data show that supplementing the diet of a pregnant mice with HFD or specific probiotics elicits cytoprotective effects on the intestinal health of adult offspring. The molecular mechanisms whereby the maternal microbiome transmits and endows enduring beneficial cell signal events in the gut of offspring is the focus of intense current research within our group.

## Data Availability

The authors confirm that the data supporting the findings of this study are available within the article, any further data can be requested from the corresponding author.
